# Phylogeography of *Schizopygopsis stoliczkai* (Cyprinidae) in Northwest Tibetan Plateau area

**DOI:** 10.1002/ece3.3452

**Published:** 2017-10-16

**Authors:** Kunyuan Wanghe, Yongtao Tang, Fei Tian, Chenguang Feng, Renyi Zhang, Guogang Li, Sijia Liu, Kai Zhao

**Affiliations:** ^1^ Key Laboratory of Adaptation and Evolution of Plateau Biota Northwest Institute of Plateau Biology Chinese Academy of Sciences Xining Qinghai China; ^2^ Laboratory of Plateau Fish Evolutionary and Functional Genomics Northwest Institute of Plateau Biology Chinese Academy of Sciences Xining Qinghai China; ^3^ Qinghai Key Laboratory of Animal Ecological Genomics Xining Qinghai China; ^4^ University of Chinese Academy of Sciences Beijing China; ^5^ Guizhou Normal University Guiyang China; ^6^ Xishuangbanna Tropical Botanical Garden Chinese Academy of Sciences Mengla China

**Keywords:** historical demography, phylogeography, Pleistocene glacial refugia, *Schizopygopsis stoliczkai*, Tibetan Plateau

## Abstract

*Schizopygopsis stoliczkai* (Cyprinidae, subfamily Schizothoracinae) is one of the major freshwater fishes endemic to the northwestern margin of the Tibetan Plateau. In the current study, we used mitochondrial DNA markers cytochrome *b* (Cyt *b*) and 16S rRNA (16S), as well as the nuclear marker, the second intron of the nuclear beta‐actin gene (Act2), to uncover the phylogeography of *S. stoliczkai*. In total, we obtained 74 haplotypes from 403 mitochondrial concatenated sequences. The mtDNA markers depict the phylogenetic structures of *S. stoliczkai*, which consist of clade North and clade South. The split time of the two clades is dated back to 4.27 Mya (95% HPD = 1.96–8.20 Mya). The estimated split time is earlier than the beginning of the ice age of Pleistocene (2.60 Mya), suggesting that the northwestern area of the Tibetan Plateau probably contain at least two glacial refugia for *S. stoliczkai*. SAMOVA supports the formation of four groups: (i) the Karakash River group; (ii) The Lake Pangong group; (iii) the Shiquan River group; (iv) the Southern Basin group. Clade North included Karakash River, Lake Pangong, and Shiquan River groups, while seven populations of clade South share the haplotypes. Genetic diversity, star‐like network, BSP analysis, as well as negative neutrality tests indicate recent expansions events of *S. stoliczkai*. Conclusively, our results illustrate the phylogeography of *S. stoliczkai*, implying the Shiquan River is presumably the main refuge for *S. stoliczkai*.

## 1 BACKGROUND

The hypotheses that landscape biogeographic features and climate change characterize the evolutionary and ecological processes act as the basis of the modern phylogeography theories (Avise, 2000; Avise et al., 1987; Mezzasalma et al., 2015). The Quaternary geographic and climatic changes across the Tibetan Plateau (TP) area shift the habitats of the endemic species, shaping the current distribution of the endemic fauna and intraspecific phylogeographic patterns (Jin & Liu, 2010; Li et al., 2015; Liu et al., 2015; Zhao et al., 2011). Therefore, the investigation of the phylogeography of the species endemic to the TP helps to achieve the following goals: to clarify the genetic structure and historical demography of the local population and to identify the relative roles of contemporary verses historical processes that have facilitated to shape the modern distribution (Avise, 2000).

As the highest and largest plateau over the world, the TP has the average altitude of ~4,500 m and the area of 2.5 × 10^6^ km^2^ (Zheng, Xu, & Shen, 2002). The formation of the TP is caused by the collision of India and Eurasia around 50–45 million years ago (Mya) (Lippert, Van Hinsbergen, & Dupont‐Nivet, 2014; Zhisheng, Kutzbach, Prell, & Porter, 2001). The uplift processes of the TP is a controversial issue (Li & Fang, 1999; Tapponnier et al., 2001). Harrison & Copeland (1992) and Lippert et al. (2014) suggest that the rapid uplifting of TP begin at about 20 Mya and the present elevation of the TP be reached by about 8 Mya. Alternatively, Cui et al. (1998) and Li et al. (2014; Hou, Li, Song, Meng, & Zhang, 2015) state that the TP reaches its maximum height before 8 Mya followed by the extensively faulting and a recent rapid uplift occurring at about 3.6 Mya which is accompanied by the formation of the largest glacier in the Northern Hemisphere. Both hypotheses admit the formation of the TP is probably a long‐standing topographic process, and the latter emphasizes its recent uplift step in Quaternary (Peng, Ho, Zhang, & He, 2006).

**Figure 1:**
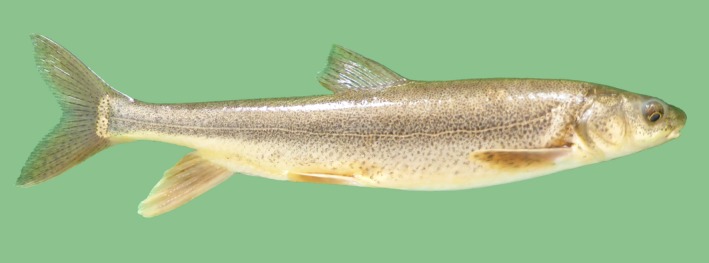
The study species, *Schizopygopsis stoliczkai* in Shiquan River

**Figure 2:**
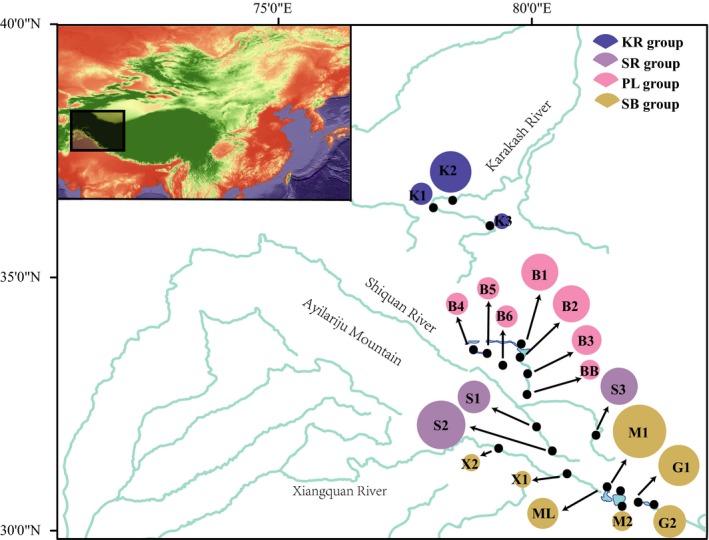
Map of sampling sites in our field surveys. The information of sampling sites referred to Table 1. The colors of the circles mean four groups defined by SAMOVA. The circle size denotes the number of observed individuals. KR, Karakash River; SR Shiquan River; PL Lake Pangong; SB, Southern Basin

*Schizopygopsis stoliczkai* (Figure 1) belongs to the subfamily Schizothoracinae (Cyprinidae). The species distributes in rivers and lakes in the northwestern of the TP (Figure 2), including rivers of Karakash, Shiquan, and Xiangquan, as well as lakes of Pangong, Manasarovar, and Kunggyu. The Shiquan River and Xiangquan River originate in the Himalayas, which are the upstream of the Indus River. The Karakash River, from the Karakoram to West Kunlun Mountain, is a tributary of the Tarim River Basin, the largest endorheic basin in the world. Because of the difficulty in sampling, *S. stoliczkai* is considered by the IUCN as a “not evaluated” species (IUCN 2010). Previous research mainly focus on the biodiversity (Mirza & Bhatti, 1999; Raghavan, Philip, Dahanukar, & Ali, 2013), the complete mitochondrial genome (Zhang, Chen, & Ding, 2016), and the morphology of *S. stoliczkai* (Kun‐Yuan et al., 2016); however, its intraspecific phylogeography is rarely studied.

It has been reported that the current phylogenetic pattern of the extant schizothoracine is driven by the environmental changes and river system transition caused by the uplift of the TP (Guo et al., 2016; Li, Tang, Zhang, & Zhao, 2016; Li et al., 2015; Zhao et al., 2011). Based on the studies of schizothoracine, we hypothesized that the current phylogenetic pattern of *S. stoliczkai* also reflects the ancient geographic and climate changes in the northwest of the TP (McQueen, Post, & Mills, 1986; Wu & Wu, 1992). To test this assumption, we collected 403 samples from all the recorded inhabits of *S. stoliczkai* in China (Figure 2) (Wu & Wu, 1992). The phylogeographic history of *S. stoliczkai* is reconstructed using both mitochondrial [cytochrome b (Cyt *b*), 16S rRNA (16S)] and nuclear [the second intron of the nuclear beta‐actin gene (Act2) genes] markers. In the current study, we successfully described the phylogeography and evolutionary history of *S. stoliczkai*. Meanwhile, the genetic diversity implicated the Shiquan River is a potential refuge center for *S. stoliczkai*.

## 2 MATERIALS AND METHODS

### 2.1 Ethics statement

All animal experiments were approved by the Animal Care and Use Committees of the Northwest Institute of Plateau Biology, Chinese Academy of Sciences and the Agriculture Department of Qinghai Province, China.

### 2.2 Sample collection

A total of 403 individuals of *S. stoliczkai* were collected during 2010 to 2015 (Table 1). The 20 localities covered all of the described distribution in China of this fish, including outflow drainages (the Xiangquan River and the Shiquan River), inflow drainage (the Karakash River), and isolate lakes (the Lake Kunggyu, the Lake Manasarovar, and the Lake Pangong) (Figure 2). In addition, nine closely related species (*Gymnocypris eckloni*,* Oxygymnocypris stewartii*,* Schizopygopsis younghusbandi*,* Schizopygopsis microcephalus*,* Schizopygopsis pylzovi*,* Gymnocypris przewalskii*,* Diptychus maculatus*,* Gymnodiptychus dybowskii*, and *Gymnodiptychus pachycheilus*) of the subfamily Schizothoracinae were referred as outgroups. All the samples were captured by seining or net casting, which were labeled and kept in 95% ethanol for the subsequently molecular analyses. All vouchered specimens were stored in the Northwest Institute of Plateau Biology, Chinese Academy of Sciences.

**Table 1:** Descriptive statistics by population site for *Schizopygopsis stoliczkai*

Basin	PC	Latitude	Longitude	Altitude	*N*	NH	*h* (D)	π	No. of sequences		
									Cyt b	16S	ACT‐2
KR	K1	36.381	77.985	3,607	10	3	0.378 (0.181)	0.00018	10	10	6
KR	K2	36.564	78.160	3,327	35	2	0.111 (0.070)	0.00005	35	35	0
KR	K3	36.200	76.771	4,046	5	1	0.000 (0.000)	0.00000	5	5	0
SR	S1	32.270	79.935	4,244	19	12	0.936 (0.037)	0.00226	19	19	19
SR	S2	31.954	80.148	4,344	50	18	0.807 (0.052)	0.00132	50	50	6
SR	S3	32.134	81.273	4,610	29	15	0.874 (0.054)	0.00185	29	29	28
PL	B1	33.633	79.817	4,270	30	4	0.193 (0.095)	0.00012	30	30	6
PL	B2	33.451	79.819	4,250	30	7	0.464 (0.111)	0.00023	30	30	6
PL	B3	33.363	79.670	4,263	18	3	0.386 (0.128)	0.00023	18	18	18
PL	B4	33.567	78.838	4,297	10	1	0.000 (0.000)	0.00000	10	10	10
PL	B5	33.504	79.017	4,301	10	2	0.200 (0.154)	0.00009	10	10	10
PL	B6	33.390	79.380	4,362	10	3	0.378 (0.181)	0.00018	10	10	6
PL	BB	33.213	79.693	4,314	8	3	0.464 (0.200)	0.00022	8	8	10
LM	ML	30.935	81.300	4,610	20	5	0.626 (0.110)	0.00069	20	20	7
LM	M1	30.743	81.593	4,595	30	9	0.811 (0.050)	0.00104	30	30	6
LM	M2	30.604	81.507	4,592	8	2	0.429 (0.169)	0.00019	8	8	6
KL	G1	30.667	82.056	4,789	30	10	0.837 (0.044)	0.00110	30	30	8
KL	G2	30.539	82.573	4,900	19	6	0.538 (0.133)	0.00102	19	19	14
XR	X1	31.116	80.770	4,374	26	6	0.815 (0.041)	0.00133	26	26	6
XR	X2	31.500	79.817	3,695	6	3	0.733 (0.155)	0.00155	6	6	14

PC, population code; *N*, population size; NH, number of haplotypes; *h*, haplotype diversity; D, standard deviation; π, nucleotide diversity; KR, Karakash River; SR, Shiquan River; XR, Xiangquan River; PL, Lake Pangong; LM, Lake Manasarovar; KL, Lake Kunggyu.

### 2.3 DNA extraction, PCR amplification, and sequencing

Total DNA was extracted from the ethanol‐fixed tissue using proteinase K digestion followed standard three‐step phenol/chloroform extraction procedure (Maniatis, 1982). Afterward, two mtDNA fragments were amplified in all 403 samples: 1,140 bp of complete Cytochrome *b* gene (Cyt *b*) and 1,118 bp of complete 16S rRNA gene (16S). The nuDNA marker (Act2) of 497 bp was amplified and sequenced in the 186 representative samples of each population (Table 1).

PCR was performed in 35 μl reactions containing 50–200 ng of DNA, 3 μl of dNTP mix (2.5 mm each), 1 U TaKaRa rTaq (TaKaRa Corp., Dalian, China), 3.5 μl 10× reaction buffer, and 0.7 μl of each primer (10 mm). All reactions were performed under the following thermal cycler conditions: denaturation at 95°C for 5 min followed by 35 cycles of 95°C for 30 s, annealing at 48–60°C for 30 s (Table 2), and extension at 72°C, 90 s for Cyt *b*, 16S, and 45s for Act2, respectively, and with a final extension at 72°C for 10 min. After visualization of the fragments using 1% agarose gel, the PCR products were sequenced from both ends using an ABI PRISM 3700 sequencing system (Beijing Tianyi Huiyuan Bioscience and Technology Incorporation, Beijing, China). All the primers were synthesized by Beijing Tianyi Huiyuan Bioscience and Technology Incorporation (Beijing, China). The sequences have been deposited in the GenBank library under the Accession Nos. KY032009–KY033062 (Appendices S1 and S2).

**Table 2:** Primer sequences used in this study

Markers	Gene	Primer name	Sequence (5′–3′)	Product length (bp)	Annealing temperature (°C)	References
Mitochondrial	Cyt *b*	L14724	GACTTGAAAAACCACCGTTG	1,140	52	Xiao, Zhang, and Liu (2001)
		H15915	CTCCGATCTCCGGATTACAAGAC			
	16S	16Sp1F	CTTACACCGAGAARACATC	1,118	48	Li et al. (2008)
		16Sp1R	CTTAAGCTCCAAAGGGTC			
Nuclear	Act2	Act18U21	GCTCCAGAAAAACCTATAAGT	~490	52	Markova et al. (2010)
		Act554L21	CTCACTGAAGCTCCTCTTAAC			

### 2.4 Sequence matrix and saturation test

The DNA sequences were assembled and analyzed by Seqman software (DNASTAR Inc., Madison, WI, USA). We conducted the alignment and the manual calibration of all the sequences using MEGA software v6.06 (Tamura, Stecher, Peterson, Filipski, & Kumar, 2013). The PHASE algorithm implemented DNAsp software was used to phase the double chromatograph peak of Act2 sequences with default parameters. Phase results were asserted by posterior value greater than 85%, and were taken for the following analysis (Garrick, Sunnucks, & Dyer, 2010; Librado & Rozas, 2009). We estimated indices of substitution saturation (I*ss* and I*ss.c*) for Cyt *b* using DAMBE (reference).

### 2.5 Population genetic structure and molecular diversity

The mitochondrial concatenated DNA (MCD) was directly used to evaluate genetic diversity, the population genetic structure, and population demographic history of *S. stoliczkai*.

Genetic diversities including nucleotide diversity (π) and haplotype diversity (*h*) (Nei, 1987) were calculated by DNAsp v5.1 (Librado & Rozas, 2009). The identical haplotype for all the sequences was obtained with the program DNAsp v5.1 (Librado & Rozas, 2009). The median‐join networks were reconstructed and visualized using Network v4.6 (Bandelt, Forster, & Röhl, 1999).

We used the software SAMOVA 2.0 (Jaffré, Joly, & Haidar, 2004) to define the groups of *S. stoliczkai* population from all the sampling locations. The spatial analysis of molecular variance (SAMOVA) was employed to search from 2 to 10 potential population units. Arlequin v3.0 (Excoffier, Laval, & Schneider, 2005) was used to estimate the pairwise genetic differentiation (*F*_ST_) values.

Demographic history was assessed using both neutral test methods and Bayesian skyline plots (BSP). The Tajima's D (Tajima, 1989) and Fu's *F*s (Fu, 1997) were calculated in neutral test to evaluate population expansion. These neutrality tests were implemented in Arlequin v3.0 (Excoffier et al., 2005) with 10,000 permutations. BSP was employed with the software BEAST v1. 7.4 to evaluate population size change over time (Drummond, Suchard, Xie, & Rambaut, 2012). Due to the small number of haplotypes (*h *=* *3), the Karakash River group was excluded from BSP analysis. BSP set up Bayesian skyline process and a random starting tree. The length of Markov Chain Monte Carlo (MCMC) chains were 50,000,000 generations, and sampling was drawn every 1,000 steps.

### 2.6 Phylogenetic analyses

Phylogenetic topologies of MCD were constructed using Bayesian inference (BI) methods implemented in MrBayes v3.2 software (Ronquist et al., 2012). The close relatives of *S. stoliczkai*,* S. younghusbandi*,* S. microcephalus*,* S. pylzovi*,* G. eckloni*,* G. przewalskii*, and *O. stewarti* were included. The root of phylogenetic tree was *O. stewarti*. The best‐fit nucleotide substitutions models, TrN + I for Cyt *b*, HKY for 16S, and HKY + I for Act2, were selected from the 88 common models using the Akaike Information Criterion by software JModelTest v2.14 (Darriba, Taboada, Doallo, & Posada, 2012). To analyze the posterior distributions from BI, we ran two concurrent MCMC analyses with one cold chain and three heated chains beginning with random trees (Li et al., 2015; Zhang et al., 2013). For MCD, MCMC chains were performed for 5,000,000 generations with a burn‐in fraction to 25%, sampled and printed every 100 steps. The convergence was assessed as the average standard deviation of the split frequencies smaller than 0.01 (Tang, Feng, Wanghe, Li, & Zhao, 2016). For Act2, we performed 2,000,000 generations of MCMC chains, sampling every 1,000 steps.

### 2.7 Divergence time estimation

The time of divergence was estimated using a lognormal relaxed clock (uncorrelated) approach in BEAST v1. 7.4 (Drummond et al., 2012). We used the Bayesian skyline process as the method of the tree prior. Due to the absence of fossil records the subfamily Schizothoracinae, the molecular clock was calibrated using the estimated split time of Schizothoracinae fishes and an accurate geological event date calibrated the molecular clock: (i) the Kunlun‐Huanghe Movement occurred 1.1–0.6 Mya (He & Chen, 2007), (ii) *D. maculatus* vs. the *G. dybowskii*‐*G. pachycheilus* clade (7.77 ± 0.51 Mya), (iii) *G. dybowskii* vs. *G. pachycheilus* (3.54 ± 0.39 Mya) (He, Chen, Chen, & Chen, 2004; Li et al., 2015). Three independent MCMC analyses were conducted with 150,000,000 generations of 20% as burn‐in.

## 3 RESULT

### 3.1 Sequence characteristics

In MCD, 56 variable sites with 37 parsimony informative sites were identified in Cyt *b* and 25 variable sites with eight parsimony informative sites were determined in 16S. The MCD contained 74 haplotypes (Appendix S1), without stop codons, insertions, or deletions. For the nuclear marker, 497 bp of Act2 segments were sequenced in 186 samples, which included eight parsimony informative sites and 13 haplotypes. The sequence information was deposited in GenBank with the Accession Nos. KY032009–KY033062 (Appendices S1 and S2). For all sites of Cyt *b*, the values of substitution saturation index I*ss* were 0.01. Given 32 OTUs, the critical I*ss.c* value is 0.757 for a symmetrical true tree, and 0.454 for an asymmetrical one. The observed I*ss* was significantly lower than both I*ss.c* values, indicating that Cyt *b* sequences did not reach saturation and were suitable for genetic analysis.

### 3.2 Genetic diversity and population genetic structure

The number of haplotypes, haplotype diversity (*h*), and nucleotide diversity (π) values within each population and in the overall population of *S. stoliczkai* are presented in Table 1. The overall nucleotide diversity (π) and the haploid‐type diversity (*h*) of concatenated mitochondrial sequences were 0.00446 and 0.894, respectively.

**Figure 3:**
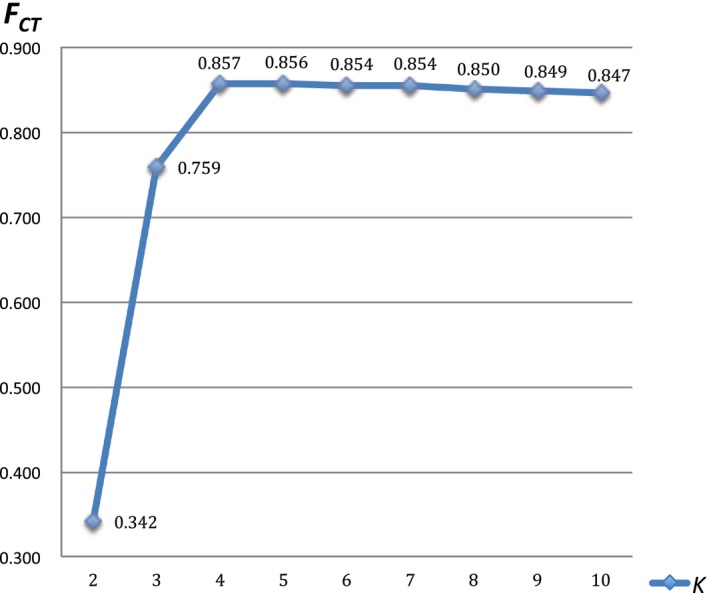
Summary of results of spatial analysis of molecular variance (SAMOVA) in *Schizopygopsis stoliczkai* populations. *K*, number of groups; *F*_CT_, variation among groups

The *F*_ST_ values between 20 populations of *S. stoliczkai* are listed in Table 3, ranging from −0.088 to 1.000. The highest significant values of group differentiation were achieved when *K* was equal to 4, indicating *S. stoliczkai* was separated as four groups (Figure 3): the Karakash River group including K1, K2, and K3, the Lake Pangong group containing B1, B2, B3, B4, B5, B6, and BB, the Shiquan River group consisting of S1, S2, and S3, and the Southern Basin group including ML, M1, M2, G1, G2, X1, and X2. The hierarchical SAMOVA analysis showed that 75.91% of the genetic variance was found among groups when *K* was equal to 3 and 85.70% of the genetic variance was reached when *K* was equal to 4 (Table 4).

**Table 3:** The *F*_ST_ values among 20 populations of *Schizopygopsis stoliczkai*

*F*_ST_	ML	M1	M2	G1	G2	X1	X2	B1	B2	B3	B4	B5	B6	BB	S1	S2	S3	K1	K2	K3
ML	0.000																			
M1	−0.006	0.000																		
M2	0.049	0.102	0.000																	
G1	0.052	0.004	0.175	0.000																
G2	−0.024	−0.010	0.056	0.026	0.000															
X1	0.140	0.057	0.227	0.074^a^	0.116	0.000														
X2	0.189	0.054	0.379^a^	0.041	0.147	0.073	0.000													
B1	0.951^a^	0.926^a^	0.981^a^	0.913^a^	0.947^a^	0.904^a^	0.953^a^	0.000												
B2	0.942^a^	0.913^a^	0.969^a^	0.907^a^	0.938^a^	0.897^a^	0.940^a^	0.011	0.000											
B3	0.933^a^	0.897^a^	0.969^a^	0.890^a^	0.927^a^	0.876^a^	0.925^a^	0.058	0.044	0.000										
B4	0.935^a^	0.893^a^	0.988^a^	0.885^a^	0.928^a^	0.868^a^	0.924^a^	−0.046	−0.034	0.022	0.000									
B5	0.931^a^	0.891^a^	0.981^a^	0.882^a^	0.924^a^	0.865^a^	0.916^a^	−0.009	−0.014	0.029	0.000	0.000								
B6	0.928^a^	0.888^a^	0.974^a^	0.880^a^	0.921^a^	0.863^a^	0.909^a^	0.015	0.000	0.034	0.000	0.000	0.000							
BB	0.922^a^	0.882^a^	0.971^a^	0.873^a^	0.915^a^	0.854^a^	0.895^a^	0.034	0.006	0.035	0.029	0.011	0.002	0.000						
S1	0.789^a^	0.781^a^	0.770^a^	0.771^a^	0.782^a^	0.751^a^	0.712^a^	0.676^a^	0.660^a^	0.579^a^	0.554^a^	0.550^a^	0.546^a^	0.524^a^	0.000					
S2	0.839^a^	0.828^a^	0.842^a^	0.820^a^	0.836^a^	0.810^a^	0.813^a^	0.751^a^	0.742^a^	0.698^a^	0.699^a^	0.697^a^	0.695^a^	0.686^a^	0.035	0.000				
S3	0.805^a^	0.795^a^	0.798^a^	0.786^a^	0.800^a^	0.770^a^	0.754^a^	0.681^a^	0.667^a^	0.600^a^	0.589^a^	0.585^a^	0.582^a^	0.566^a^	−0.023	0.032	0.000			
K1	0.935^a^	0.900^a^	0.977^a^	0.893^a^	0.929^a^	0.877^a^	0.919^a^	0.971^a^	0.952^a^	0.952^a^	0.980^a^	0.970^a^	0.961^a^	0.957^a^	0.546^a^	0.607^a^	0.578^a^	0.000		
K2	0.964^a^	0.937^a^	0.990^a^	0.932^a^	0.962^a^	0.925^a^	0.969^a^	0.982^a^	0.970^a^	0.974^a^	0.991^a^	0.987^a^	0.983^a^	0.982^a^	0.707^a^	0.693^a^	0.703^a^	0.004	0.000	
K3	0.930^a^	0.891^a^	0.985^a^	0.883^a^	0.922^a^	0.864^a^	0.897^a^	0.977^a^	0.955^a^	0.957^a^	1.000^a^	0.986^a^	0.973^a^	0.969^a^	0.487^a^	0.580^a^	0.537^a^	−0.084	−0.088	0.000

The population code numbers see Table 1.

Population code follows Table 1.

a *p* <* *.001.

**Table 4:** Hierarchical analysis of molecular variance (AMOVA) based on MCD *Schizopygopsis stoliczkai*

*K*	Source of variation	*df*	Sum of squares	Variance components	Percentage of variation	*p* value
3	Among groups	2	1,377.25	5.64	75.91	.00
	Among populations within regions	17	300.16	0.88	11.84	.00
	Within populations	383	349.30	0.91	12.25	.00
	Total	402	2,026.73	7.44		
4	Among groups	3	1,653.74	5.65	85.70	.00
	Among populations within regions	16	23.67	0.03	0.47	.00
	Within populations	383	349.30	0.91	13.83	.00
	Total	402	2,026.73	6.59		

*K*, number of groups; *df*, degrees of freedom.

### 3.3 Haplotype network and phylogeographic structure

**Figure 4:**
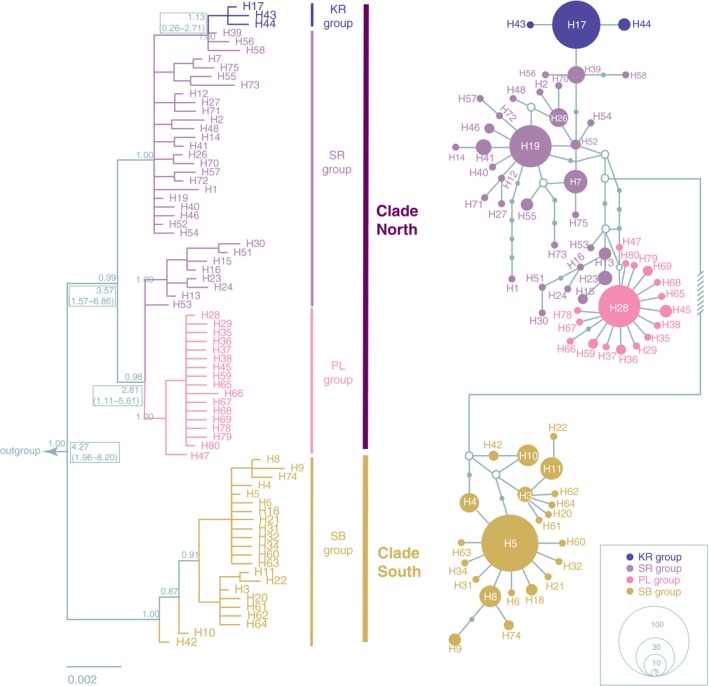
The BI tree (left) and the corresponding median‐joining network (right) assessed by 16S–Cyt b combined sequences. The numbers above the branches indicate the Bayesian posterior probabilities. Mean time to the TMRCA with 95% highest posterior density (95% HPD) in parenthesis for the key nodes is given in the boxes (Mya). The details of those haplotypes see Appendix S1. The detail of outgroups see Appendices S1 and S3. In the network, the circle size denotes the number of observed individuals, scaling in the lower. The small white circles represent missing intermediate haplotypes. KR, Karakash River; SR, Shiquan River; PL, Lake Pangong; SB, Southern Basin

Based on the MCD, the phylogenetic analysis showed *S. stoliczkai* was comprised of two distinct lineages (Figure 4, left). All of the specimens distributed in the north of Ayilariju Mountains (Figure 2) belonged to the clade North (clade N), and the rest were grouped into clade South (clade S). In the clade N, the Karakash River and the Lake Pangong groups were clustered in a monophyly, respectively. The Southern Basin group was a paraphyly. The clade S included all the populations from the Southern Basin group. The detailed information of outgroups in the BI tree was shown in Appendices S1 and S3.

**Figure 5:**
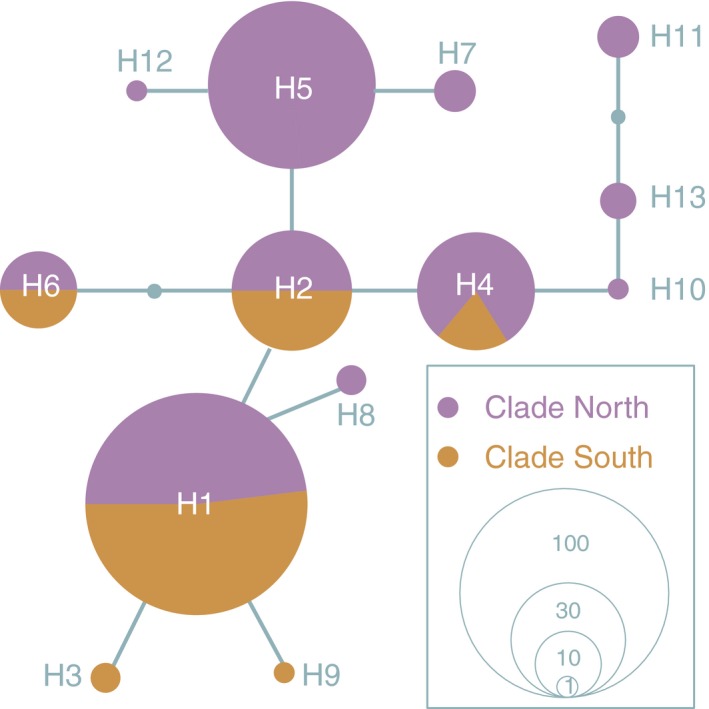
The median‐joining network derived from Act2 segments. The lineages represented by different colors and the circle size denating the amount of observations are labeled in lower right corner

The haplotype network of MCD for *S. stoliczkai* also indicated the existence of four groups (Figure 4, right), which was in line with the tree topologies. The distance between clades N and S was nine mutational steps. The haplotype divergence within the groups of the Shiquan River, the Karakash River, the Lake Pangong, and the Southern Basin were 16, 3, 4, and 10 mutational steps, respectively. The Shiquan River group was considered as the ancestor of the other three groups as they had the highest genetic diversity. Moreover, the Lake Pangong group and the Southern Basin group displayed star‐like shapes, indicating recent population expansions. Some intermediate haplotypes were missed in the network, which was probably resulted from the population extinction or depression. However, the nuclear gene network (Figure 5) and BI tree (Appendix S2) could not show such topology of the clade N and clade S.

### 3.4 Divergence dating and historical demography

The molecular clock (Figure 4, boxes) predicted the divergence between the clade N and clade S occurred at about 4.27 Mya (95% HPD = 1.96–8.20 Mya). The split time between the Lake Pangong group and the Shiquan River group was at 2.81 Mya (95% HPD = 1.11–5.61 Mya). Divergent time between the Karakash River group and the Shiquan river group was at 1.13 Mya with 95% HPD of 0.26–2.71 Mya.

**Figure 6:**
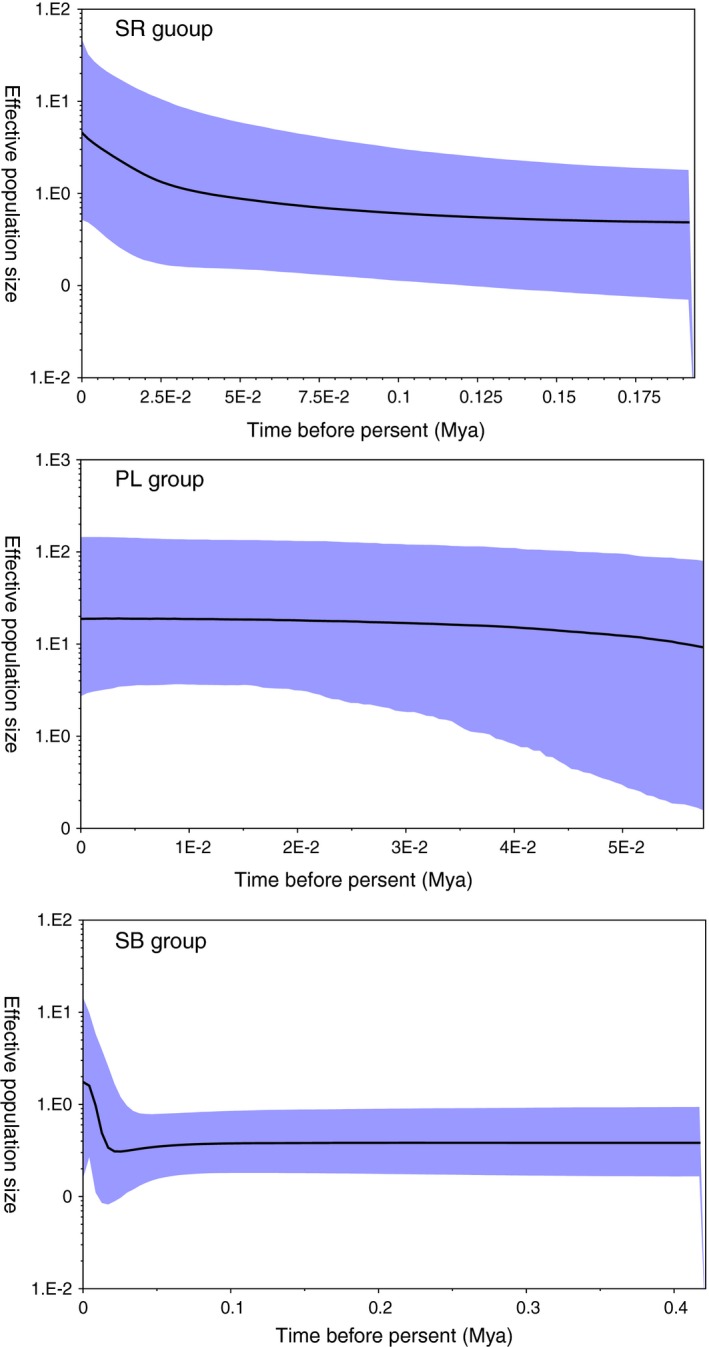
Bayesian skyline plots (BSPs) estimated by BEAST of *S. stoliczkai* in three allopatric clades. The *X*‐axis indicates time in millions of years before present. The *Y*‐axis shows effective population size as *N*_e_ on a log scale. The shaded areas depicted in blue exhibit 95% HPD credibility interval, and the solid line in the middle of the blue indicates the median of population size. SR, Shiquan River; PL, Lake Pangong; SB, Southern Basin

In neutrality test (Table 5), negative Fu's *F*s values and Tajima's D values indicated that the quick expansion occurred in all the groups. BSP results (Figure 6) were consistent with neutrality test, supported the population expansion. Within clade N, the moderate demographic expansion happened in the Shiquan River group in early 0.03 Mya, and the Lake Pangong group maintained stability during the last 0.05 Mya (Figure 6). In clade S, a pronounced demographic and relatively recent expansion happened the Southern Basin group in 0.025 Mya (Figure 6).

**Table 5:** Genetic diversity, neutrality tests, as well as number of specimens and haplotypes of each clade

Group	No. of specimens	No. of haplotypes	π	*h* (*SD*)	Neutrality test	
					Tajima's D	Fu's *F*s
KR Group	50	3	0.00007	0.153 (0.067)	−1.16,435	−1.828*
SR Group	98	32	0.00166	0.856 (0.031)	−1.66,138*	−17.715**
PL Group	116	17	0.00016	0.301 (0.057)	−2.45,978**	−24.258**
SB Group	139	22	0.00105	0.774 (0.031)	−1.61,657*	−9.161**
Clade North	264	52	0.00246	0.816 (0.018)	−1.14,852	−21.952**

KR, Karakash River; SR, Shiquan River; PL, Lake Pangong; SB, Southern Basin.

π denotes nucleotide diversity; *h* (*SD*) is haplotype diversity with standard deviation.

Significant pairwise differences: *p <* *.05, **p <* *.01.

## 4 DISCUSSION

The current study was the first intraspecific phylogeographic study of *S. stoliczkai*. We conducted the intensive sampling to construct the phylogeographic relationship among different populations of *S. stoliczkai*. The results indicated the population structure and demography of *S. stoliczkai* were probably related to the glacial cycles and the uplift of the TP.

### 4.1 Phylogeography and population expansion of *S. stoliczkai*

Geological movement and climatic fluctuations play an essential role in the phylogeography pattern of many species endemic to the TP (Jin & Liu, 2010; Li et al., 2015, 2016; Liu et al., 2015; Zhang et al., 2013; Zhao et al., 2011). In our study, the estimation of the divergence time for *S. stoliczkai* (Figure 4) was broadly consistent with these studies. The age of the most recent ancestor of clade S and clade N was estimated at about 4.27 Mya, following by the divergence between the Lake Pangong group and the Shiquan River group as well as between the Karakash River group and the Shiquan River group. The molecular date estimates obtained in the present study corresponded well with the hypothesis that the formation of the QTP underwent the upheaval, flaunting, and the recent uplift (Hou et al., 2015; Li et al., 2014). Meanwhile, both in mtDNA (Figure 4) and nuclear sequences (Appendix S2), seven populations within clade S shared the common haplotypes and lacked the population structures, which were probably results from the repeated separation and the connection of drainages in the South Basin due to the glacial–interglacial cycles and/or tectonic movement during the Quaternary period (Bingyuan, Fubao, Yichou, & Qingsong, 1982; Yang & Scuderi, 2010; Zou & Dong, 1992).

Multiple analyses, including BSP, star‐like network and negative neutrality tests all supported the population expansion in *S. stoliczkai*. The population expansion had been reported in other schizothoracine fishes, including *Diptychus maculatus* (Li et al., 2016), *Gymnodiptychus dybowskii* (Li et al., 2015), and *Schizothorax nukiangensis* (Chen, Du, & He, 2015). In the current study, we found the *S. stoliczkai* experienced the population expansion from 0.05 to 0.025 Mya. Similarly, Li et al. showed *Diptychus maculatus* that distributed closely to *S. stoliczkai* also underwent the population expansion at about 0.025 Mya (Li et al., 2016), which was in good line with our results. Therefore, we speculated that the population expansion of *S. stoliczkai* was possibly benefited from the humid and warm climate during the interglacial period (Yang & Scuderi, 2010).

### 4.2 Multiple glacial refugia in the northwest edge of the Tibetan Plateau

Many species endemic to the TP area has multiple glacial refugia, such as *Gymnocypris chilianensis* (Zhao et al., 2011) and *Nanorana parkeri* (Liu et al., 2015). In the current study, we found the separation of clade N and S (4.27 Mya) was much earlier than the onset of the ice age (2.6 Mya) (Gribbin, 1983), which implicated that the clade N and S occupied different refugia during Quaternary glaciations. Moreover, a series of geochronological researches proved the existence of melting water in the upstream of the Indus River in the ice age (Owen, Caffee, Finkel, & Seong, 2008; Schäfer et al., 2002; Shi, 2002; Zhou, Wang, Wang, & Xu, 2006), which was likely to be the habitat for *S. stoliczkai* during ice age. However, it was difficult to predict the accurate refuge for clade S due to the lack of the population structure. The highest genetic diversity of the Shiquan River group (Tables 1 and 5; Graham, VanDerWal, Phillips, Moritz, & Williams, 2010) implied that the Shiquan River was probably the refuge for clade N.

Conclusively, we clearly depicted the population structure of *S. stoliczkai*, with four subdivisions corresponded well to the separated drainages. The results also implicated that *S. stoliczkai* occupied multiple refugia during the Quaternary glaciations in the different habits. Based on the population demography analysis, we speculated that the population of *S. stoliczkai* was expanded, which was highly likely due to the warm and humid climate during the interglacial period.

## CONFLICT OF INTEREST

None declared.

## AUTHOR CONTRIBUTION

All co‐authors designed the experiment; KW, YT, RZ, and GL performed the experiment; KW and YT analyzed and interpreted the data; KW and KZ wrote the paper; CF, YT, RZ, and GL worked in the field sampling. All co‐authors participated in the scientific discussions and commented on the manuscript.
