# Supplementary figures and images for: Phylogeography of Schizopygopsis stoliczkai (Cyprinidae) in Northwest Tibetan Plateau area
Source: Ecol Evol. 2017 Oct 16;7(22):9602–12. doi: 10.1002/ece3.3452 (PMC5696390; doi:10.1002/ece3.3452)

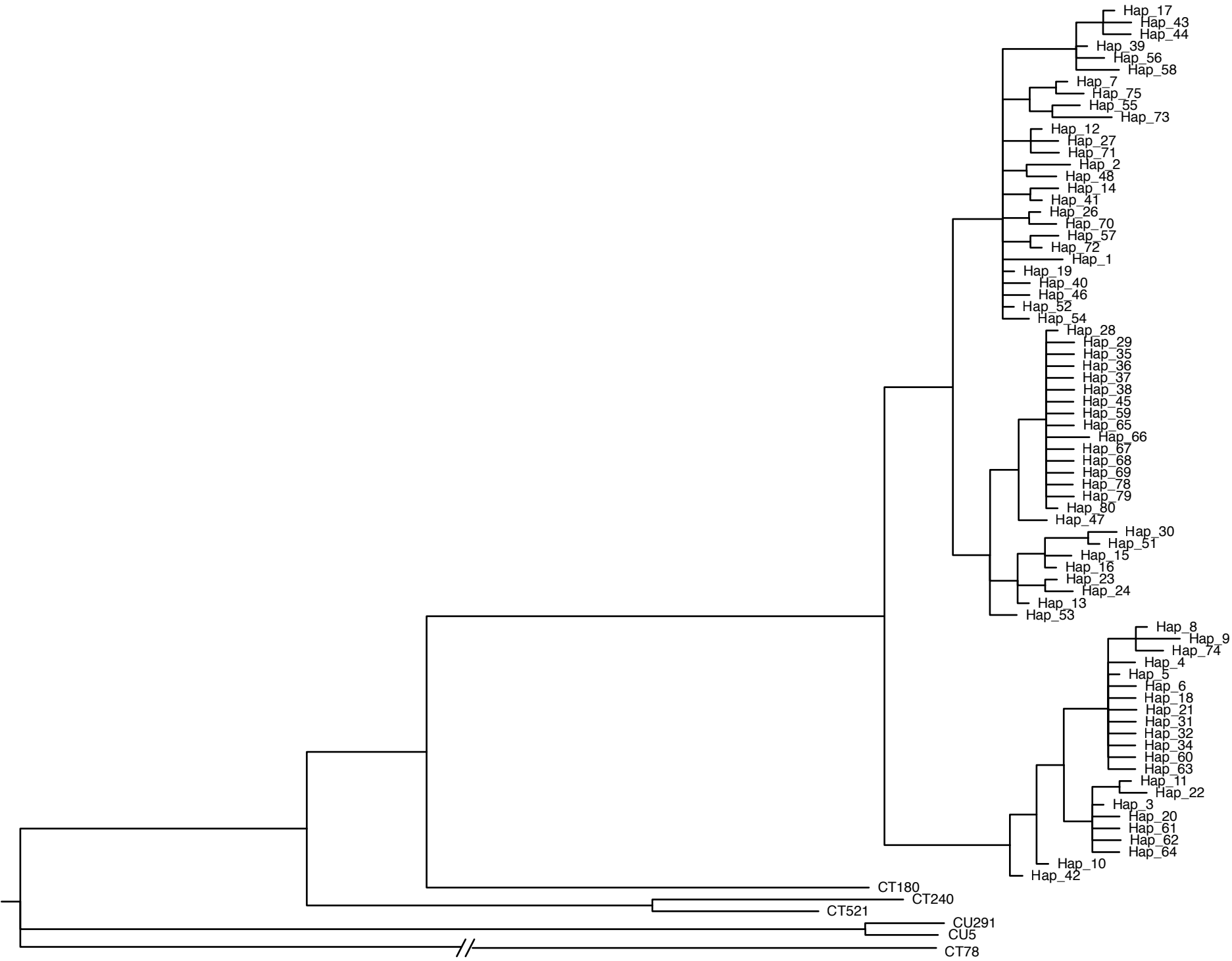

0.006

Supplement: Supplementary file 3 [file ECE3-7-9602-s003.pdf]
